# Mechanical-Stretch of C2C12 Myoblasts Inhibits Expression of Toll-Like Receptor 3 (TLR3) and of Autoantigens Associated with Inflammatory Myopathies

**DOI:** 10.1371/journal.pone.0079930

**Published:** 2013-11-04

**Authors:** Rong Chen, Liqiang Feng, Mo Ruan, Xinghui Liu, Sahil Adriouch, Hua Liao

**Affiliations:** 1 Department of Anatomy, Southern Medical University, GuangZhou, China; 2 Department of Anatomy, NingXia Medical University, NingXia, China; 3 The Affiliated Orthopedic Hospital, KunMing General Hospital of ChengDu Military Command, KunMing, China; 4 Inserm U905, University of Rouen, Institute for Research and Innovation in Biomedicine (IRIB), Normandy, France; Universidad Europea de Madrid, Spain

## Abstract

Recent studies in patients suffering from inflammatory autoimmune myopathies suggested that moderate exercise training improves or at least stabilizes muscle strength and function without inducing disease ﬂares. However, the precise mechanisms involved in this beneficial effect have not been extensively studied. Here we used a model of *in vitro* stretched C2C12 myoblasts to investigate whether mechanical stretch could influence myoblast proliferation or the expression of proinflammatory genes. Our results demonstrated that cyclic mechanical stretch stimulated C2C12 cell cycling and early up-regulation of the molecules related to mechanical-stretch pathway in muscle (calmodulin, nNOS, MMP-2, HGF and c-Met). Unexpectedly, mechanical stretch also reduced the expression of TLR3 and of proteins known to represent autoantigens in inflammatory autoimmune myopathies (Mi-2, HRS, DNA-PKcs, U1-70). Interestingly, stimulation or inhibition of calmodulin, NOS, HGF or c-Met molecules *in vitro* affected the expression of autoantigens and TLR3 proteins confirming their role in the inhibition of autoantigens and TLR3 during mechanical stretch. Overall, this study demonstrates for the first time that mechanical stretch could be beneficial by reducing expression of muscle autoantigens and of pro-inflammatory TLR3 and may provide new insight to understand how resistance training can reduce the symptoms associated with myositis.

## Introduction

Skeletal muscles are quite responsive to mechanical stress. Accumulating evidence suggests that a number of cellular components mediating mechanical transduction are involved in regulating satellite cell activation, initiating myogenic differentiation, and the development of skeletal muscles [1-3]. The molecular mechanism of satellite cell activation and proliferation triggered by mechanical stress has been identified, and correspond to a cascade of events initiated by calcium-calmodulin complex formation [4], nitric oxide (NO) radical production [5,6], matrix metalloproteinases (MMPs) activation [7], liberation of hepatocyte growth factor (HGF) with associated extracellular segment of proteoglycans, and the subsequent presentation to the receptor c-Met to generate a signal for satellite cell activation [8-10]. 

In patients suffering from inflammatory myopathies exercise training is, at present, not recommended, as physical activity was believed to potentially increase the inflammation in affected muscles. However, recent studies in patients suffering from polymyositis (PM) and dermatomyositis (DM) support the notion that moderate exercise training (e.g., a 5-days-a-week for 12-week resistance training home program) improves or at least stabilizes muscle strength and functional ability without inducing disease ﬂares [11]. Resistance training has been proven to restore muscle function by reducing inflammation and tissue fibrosis, improving metabolic homeostasis through the reduced expression of proinflammatory and profibrotic gene networks, and by increasing the expression of oxidative metabolism genes [12]. Conceivably, moderate exercise by inducing muscle strain could induce up-regulation of the key molecules involved in the response to mechanical-stretch culminating in the activation of satellite cells and in partial muscle repair. However, the consequences of up-regulation of these molecules on the expression of proteins known to represent potential autoantigens or on proinflammatoy genes have not yet been explored. As concomitant up-regulation of toll-like receptors (TLRs) or of potential autoantigens could potentially aggravate the disease by stimulating cytokines and chemokines production, as well as the activation of autoimmune T cells [13-17], we thought here to explore how the induction of the mechanical-stretch pathway could influence their expression.

This is of particular importance in chronic inflammatory situations, as regenerating muscle cells are considered to be the main source of autoantigens and express higher levels of TLRs as compared to mature differentiated myotubes. Consistent with this, cultured myoblasts *in vitro* express high levels of autoantigens and of TLR3 and TLR7 that are strikingly down regulated as cells differentiate into myotubes [15,16]. We explored here the consequences of mechanical stress regarding myoblast proliferation and expression of autoantigens and of TLR3 and TLR7 in an *in vitro* model of mechanical stretch. For that, C2C12 myoblasts were cultured and submitted to controllable mechanical-stretch during cyclic period using the FlexCell system. Interestingly, our results show that mechanical-stretch stimulated, as expected, cell cycling but remarkably also reduced the expression of TLR3 and of proteins known to represent potential autoantigens. Hence, these data may help to better understand the newly recognized beneficial role of moderate exercise in patients suffering from inflammatory myopathies.

## Materials and Methods

### Cell culture and induction of mechanical-stretch

C2C12 cells (ATCC, USA) were plated in Dulbecco’s modified Eagle’s medium Nutrient Mixture F-12 (DMEM/F12, Thermo), supplemented with 10% fetal bovine serum (FBS), 100units/ml penicillin, and 100mg/ml streptomycin sulfate in a 5% CO_2_-humidified chamber (Heraeus, Germany) at 37°C. Cells were grown to approximately 70-80% confluence and used for next experiments.

For the induction of mechanical stretch, C2C12 cells were resuspended in the growth medium (GM) defined above and plated onto type I collagen-coated flexible-bottom six well plates (BioFlex plates collagen I, FlexCell International Corporation, Hillsborough, NC, USA) and incubated at 37°C in a CO_2_ incubator for 24h before applying mechanical strain. The number of seeded cells was limited to 1×10^5^ cells/well, to keep cell confluency at less than 45% during the first 24h of culture. Cells were then subjected to cyclic strain of 10% lengthening, at 0.25Hz frequency for 2h per day, or of 15% lengthening, at 1.0Hz frequency, for 1h per day, using a computer-controlled vacuum stretch apparatus (FX-5000 Tension System, FlexCell International Corporation). In control cultures, cells were growth in parallel with identical experimental conditions but were left unstretched. C2C12 cells were then assessed 2, 4 or 6d after initiation of cyclic strain to evaluate their cycling rate or the expression of molecules at study. As we noticed signs of differentiation to myotubes after day 4, and to avoid any confounding effect due to cell differentiation, myoblasts were always stretched for less than 4 day. 

### Cell cycle analysis using flow cytometry

For cell cycle analysis, stretched and unstretched C2C12 cells were collected and fixed with 80% ethanol at 4°C for 24h, and further incubated overnight at 4°C with 1ml of a propidium iodide (PI) staining mixture according to the manufacturer’s protocol (kit from GENMED Scientifics INC., USA). After staining, 10^5^ cells were analyzed by flow cytometry using a FACS Calibur flow cytometer (BD, USA). The relative DNA proliferation index [DPI = (S%+G_2_/M%)/(S%+G_2_/M%+G_0_/G_1_%)] was used to evaluate the cycling rate of C2C12 cells.

### Use of agonist or antagonist to interfere with key molecules involved in the response to mechanical-stretch

In some experiments, cells were treated with agonists or antagonists to interfere with the key molecules involved in the response to mechanical-stretch. For that, C2C12 cell cultures were washed at 24h post-plating with serum-free DMEM and then treated during 4h with the following agonist or antagonist molecules: calcium ionophore A23187 [3µM, Calbiochem-Novabiochem (La Jolla, CA)]; ethylene glycol tetra-acetate (EGTA, 1.8mM, Tocris, British); calmidazolium chloride (R24571, 0.3uM, Santa Cruz, USA); recombinant murine HGF (10ng/ml, R&D); rabbit polyclonal anti-HGF (2ug/ml, abcam); recombinant murine HGF R (c-Met, 1ug/ml, LEINCO); rabbit polyclonal anti-c-Met (2ug/ml, Santa Cruz, USA); recombinant human MMP-2 (CF, 10ng/ml, R&D, USA); MMP-2 inhibitor I (250ng/ml, Santa Cruz, USA); sodium nitroprusside dehydrate (SNP, 8ug/ml, Sigma); or L-NG-Nitroarginine Methyl Ester (L-NAME, 10uM, Santa Cruz, USA ). Cells were then washed again with serum-free DMEM, and harvested for analysis by qRT-PCR or Western Blot. C2C12 cells cultured in serum free DMEM for 4h but without the addition of agonist or antagonist were taken as controls.

### RNA isolation and analysis by quantitative real-time RT-PCR

24 hours after cessation of stretch, or 2 hours after treatment with agonists or antagonists, total RNA was extracted from C2C12 cells. For that, cells were harvested in 1ml of TRIzol® (Invitrogen) and total RNA were purified according to manufacturer’s instructions. 1μg of total RNA was then used for reverse transcription (RT) with commercially available kit (RevertAid First Strand cDNA Synthesis Kit, Fermentas). Real-time polymerase chain reaction (PCR) was performed in triplicate with an ABI StepOne Plus system (Applied Biosystems, USA) and a fluorescence-labeled SYBR Green/ROX qPCRMaster Mix kit (Fermentas) for the following genes: Mi-2, HARS, DNA-PKcs, U1-70, TLR3 and TLR7, and with glyceraldehyde-3-phosphate dehydrogenase (GAPDH) taken as an endogenous control (primer sequences and sizes of amplicons are listed in table 1). The results were analyzed with SOS2.1 software (Applied Biosystems). Expression of the genes was calculated from the accurate threshold cycle (Ct), which is the PCR cycle at which an increase in fluorescence from SYBR Green probes above the baseline signal can first be detected. The Ct values for GAPDH were compared with those from Mi-2, HARS, DNA-PKcs, U1-70, TLR3 and TLR7, and in each well to calculate ΔCt. Data of the treated conditions were expressed relative to the signal obtained for the average of the untreated controls by the ΔΔCt calculation. The triplicate ΔΔCt values for each sample were averaged. 

**Table 1:** Primer sequences for qRT-PCR.

**Genes**	**Sequence (5’→3’)**	**Amplicon size**
Mi-2	For CCCGAGGAGTGTGGA ACTA	62bp
	Rev CCCTACCACCCTAGCCAAG	
HARS	For GATGGGATGTTTGCTGTCTG	114bp
	Rev TCCCACCATCTCATTCTTCA	
DNA-PKcs	For ATATCCTTGGCAGGACTTGG	92bp
	Rev AGGTCCTCTCGGAGACAGAA	
U1-70	Fo r GACAGCAGGAAGTGGAGACA	91bp
	Rev GCCACGAACAGAGTCTTGAA	
TLR3	For TCTGTTTGCGAAGAGGAATG	114bp
	Rev AATTCCGAGATCCAAGTGCT	
TLR7	For TTGCAACTGTGATGCTGTGT	106bp
	Rev TTTGTGTGCTCCTGGACCTA	
GAPDH	For CAATGTGTCCGTCGTGGATCT	124bp
	Rev GTCCTCAGTGTAGCCCAAGATG	

### Western blot analysis

Cells were collected, washed with ice-cold PBS and protein extraction was performed according to the manufacturer's protocol (KeyGEN; China). Protein concentrations were evaluated using a BCA assay kit (Solarbio, BeiJing, China). Equal amounts of proteins were electrophoresed on 6-12% SDS-polyacrylamide gel and transferred to Immobilon P membrane (Millipore, USA). Membranes were blocked in 5% non-fat dried milk in Tris-buffered saline/Tween-20 (TBS-T: 20mM Tris, pH7.5, 150 mM NaCl, 0.05% Tween-20) for 1h at room temperature. The following rabbit or goat polyclonal antibody were used for detection: anti-Mi-2 (1:2000, abcam); anti-HARS (1:400, abcam); anti-DNA-PKcs (1:100, santa cruz); anti-U1-70 (1:100, santa cruz); anti-TLR3 (1:800, abcam); anti-TLR7 (1:100, santa cruz); anti-calmodulin (1:100, santa cruz); anti-nNOS (1:100, santa cruz); anti-HGF (1:400, abcam); anti-c-Met (1:100, santa cruz); anti-MMP-2 (1:400, abcam); anti-GAPDH (1:3000, KANGCHEN, China). Primary polyclonal antibodies were incubated for 20h at 4°in 5% non-fat dried milk in TBS-T. The membrane was then washed three times in TBS-T and incubated for 1h at room temperature with a 1:4000 horseradish peroxidase conjugated goat or donkey secondary antibody to rabbit or goat IgG (santa cruz, USA), in 5% non-fat dried milk in TBS-T. After three washes in TBS-T, the protein bands were visualized by enhanced chemiluminescence (ECL) detection reagents (Applygen Technologic Inc., China). Immunoreactive bands were scanned, and densitometric values were analyzed with Quantity One (Bio-Rad, USA). Relative expression of each immunoreactive band was calculated by comparison with GAPDH.

### Statistical analysis

All data are expressed as mean ± standard deviation (SD). One-way ANOVA was used for multiple comparisons (Duncan’s multiple range test) using SPSS ver.13.0 software. *P* values<0.05 were considered as statistically significant. 

## Results

### Mechanical-stretch promotes cell cycling of C2C12 myoblasts *in vitro*

**Figure 1:**
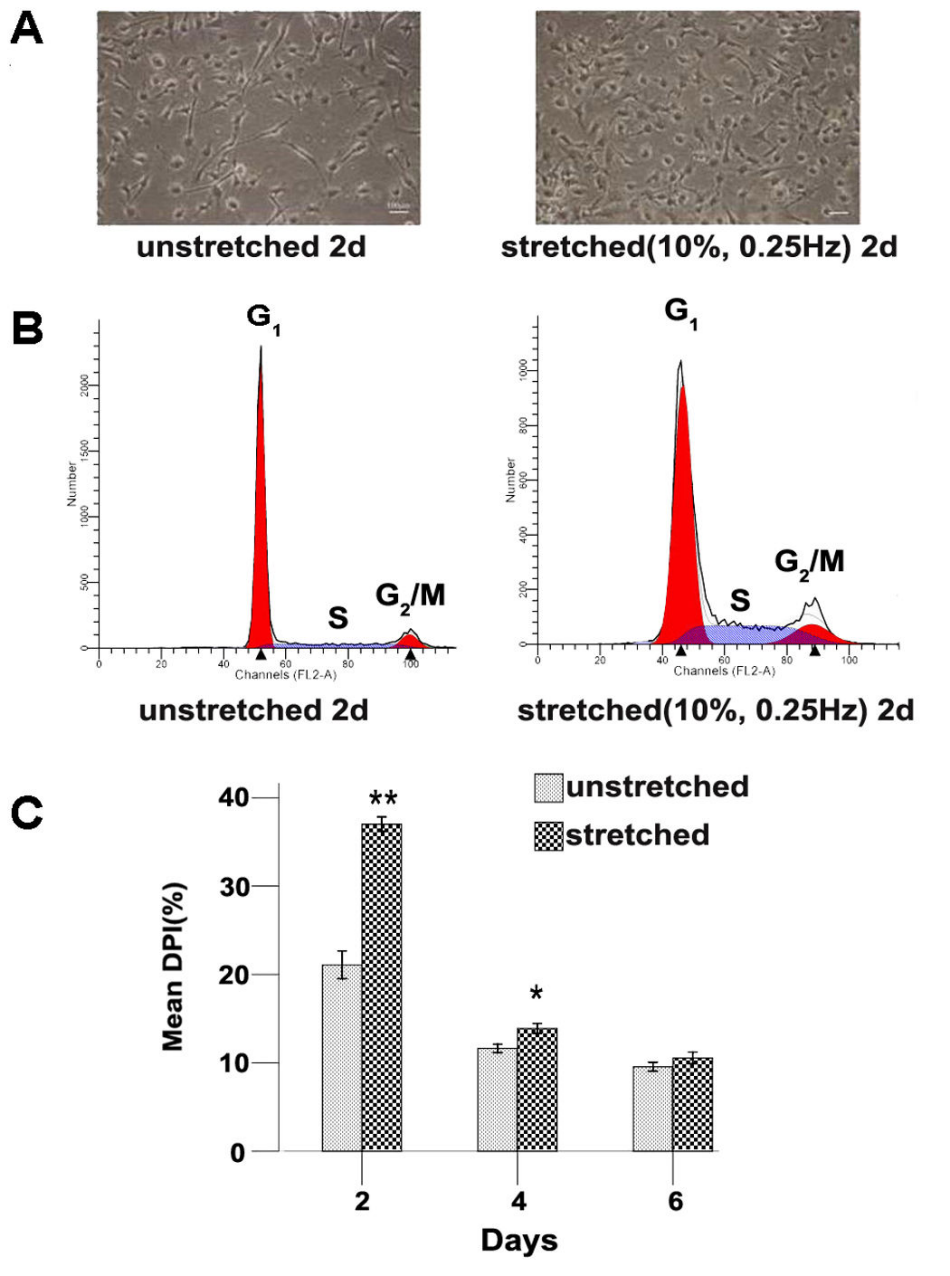
Mechanical-stretch stimulates cell cycling of C2C12 cells. (**A**) Phase contrast microscopical analysis of C2C12 myoblasts stretched or not during 2d. Cell number was higher in stretched groups (10%, 0.25Hz) than in unstretched controls. (**B**) Flow cytometric analysis of the percentage of cells in G_1_, S, or G_2_/M phases of the cell cycle. (**C**) Statistical analysis of the relative DNA proliferation index (DPI) of stretched (10%, 0.25Hz) or unstretched C2C12 myoblasts. Values represent mean ± SD (*n*=3 per group). *p* values were determined by independent-sample *t* tests (**p<0.001, *p<0.05).

Cyclic mechanical stretch has been suggested to induce the activation of primary satellite cells and of cultured myoblast *in vitro*, to accelerate their entry into the cell cycle, and to inhibit their differentiation into myotubes [18,19]. We thought here to apply mechanical-stretch and to evaluate its effect on the C2C12 myoblast model using the FlexCell system. The first purpose of this study was to set the experimental conditions and to compare two distinct stretch protocols that have been used elsewhere to induce mechanical-stretch [18,19]. These protocols either consist on the application of 15% lengthening, with a 1.0Hz frequency during 1h per day (15%, 1Hz) or instead of 10% lengthening, with a 0.25Hz frequency during 2h per day (10%, 0.25Hz). Results showed that the application of the 15%, 1.0Hz protocol induced conspicuous cell detachment and cell loss even at the earliest time point analyzed (data not shown). This is consistent with the previous suggestion that ≧15% lengthening mechanical-stretch during 1 to 5 days could cause signiﬁcant injury to muscle cells [20,21]. Therefore, this protocol was not used further in the following experiments. Instead, application of the 10%, 0.25Hz protocol allowed normal cell attachment (Figure 1A) and, further, stimulated the entry of C2C12 cells into cell cycle (Figure 1B). Indeed, analysis of the cell cycle by flow cytometry revealed obviously higher numbers of cell in the S or G_2_/M phase in stretched cells. This was particularly demonstrable at day 2 as compared unstretched control cells where the calculated relative mean DNA proliferation index (DPI) was significantly higher than that of unstretched cells, i.e., 37.2% vs. 21.1% respectively, *p*<0.001 (Figure 1C). At day 4, mean DPI of stretched cells was still higher than in the controls cells (i.e., 13.9% vs. 9.6%, *p*<0.05). However, no significant difference was detected using this assay after day 4, although the mean DPI of stretched cells at day 6 was still slightly higher than in the control cultures, i.e., 11.6% vs. 10.6% (Figure 1C). Therefore, we focused the rest of our analyses on cells stretched during 2 days where the maximum biological effect could be demonstrated. Also, at this time point, no apparent signs of differentiation into myotubes could be observed while this was clearly the case for cultures obtained on later time points (data not shown). Hence, our data demonstrate so far that cyclic mechanical-stretch of cultivated undifferentiated C2C12 myoblast stimulate their prompt entry into the proliferative phase in agreement with previous data obtained with primary satellite cells and of myoblasts cultured *in vitro* [18,19].

### Strain increases the expression of characteristic proteins involved in the response to mechanical stretch in C2C12 myoblasts

**Figure 2:**
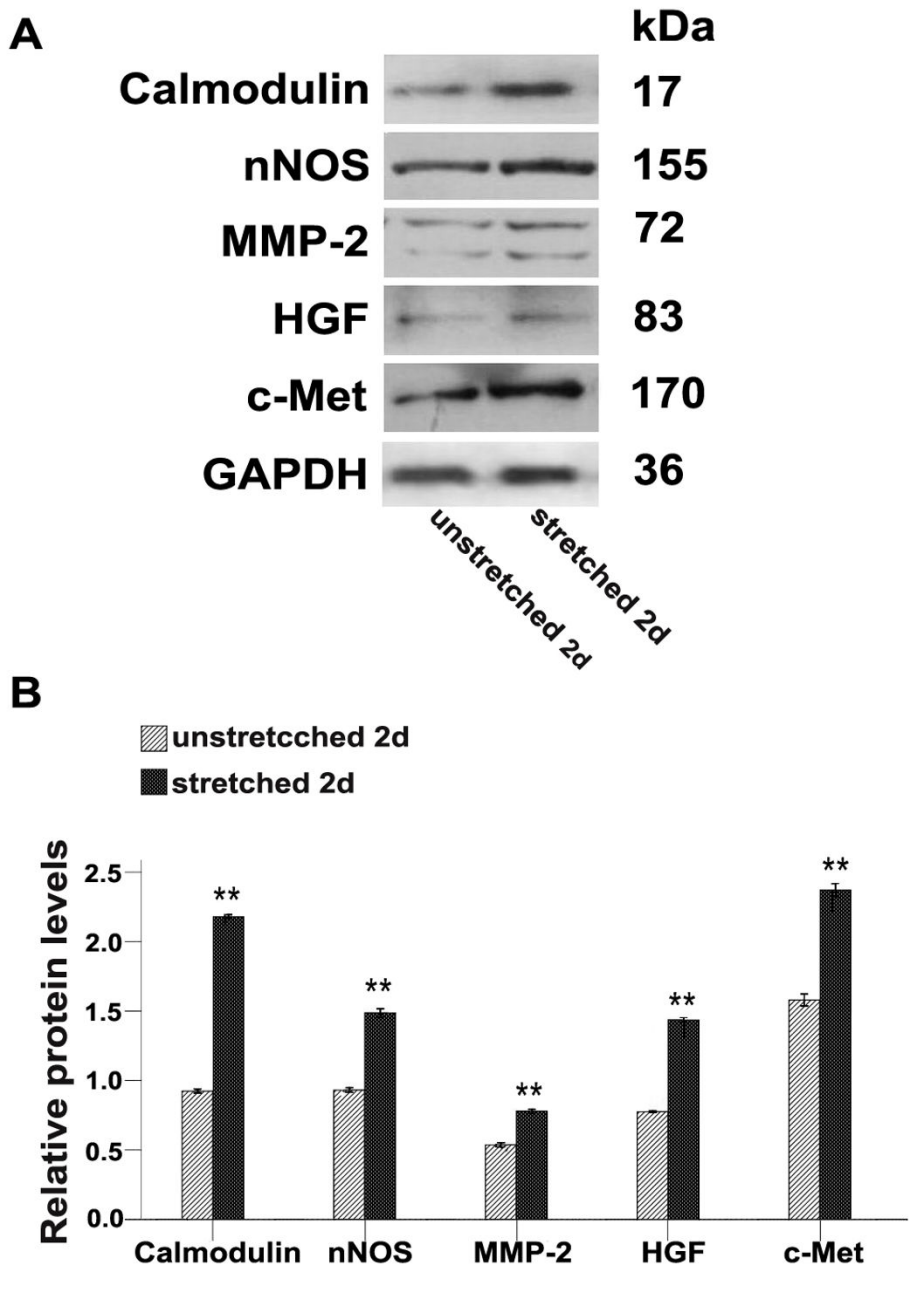
Mechanical-stretch increases the expression of proteins related to the mechanical-stretch pathway in C2C12 cells. (**A**) Western blots analysis of calmodulin, nNOS, MMP-2, HGF and c-Met in 2d stretched and unstretched C2C12 cells. (**B**) The relative band intensities were normalized to the level of GAPDH and analyzed with Quantity One software. One-way ANOVA was used for multiple comparisons. All data are presented as mean ± SD (n=3). (**p<0.001).

We next evaluated whether straining C2C12 myoblasts *in vitro* can induce the up-regulation of the characteristic key molecules that have been linked to the mechanical-stretch pathway. For that, C2C12 cells were stretched during 2 days using the 10%, 0.25Hz protocol and protein extracts were then analyzed by Western blots to evaluate the levels of calmodulin, nNOS, MMP-2, HGF and c-Met proteins. Results demonstrated that mechanical-stretch induces a significant elevation of calmodulin, nNOS, MMP-2, HGF and c-Met protein levels by a factor of, respectively, 2.4-, 1.6-, 1.5-, 1.9-, and 1.5-fold, relative to their levels in unstretched cells (Figure 2). Of notes, our results did not show substantial difference in the levels of these proteins as compare to unstretched controls when mechanical strain was apply during 4 days (data not shown). Again, this suggested that mechanical stretch induce more prominent effects on undifferentiated C2C12, on the early phase of the culture, than on later time points where signs of myotubes differentiation could be detected.

### Mechanical-stretch down regulates the expression of autoantigens and TLR3 in C2C12 myoblasts

**Figure 3:**
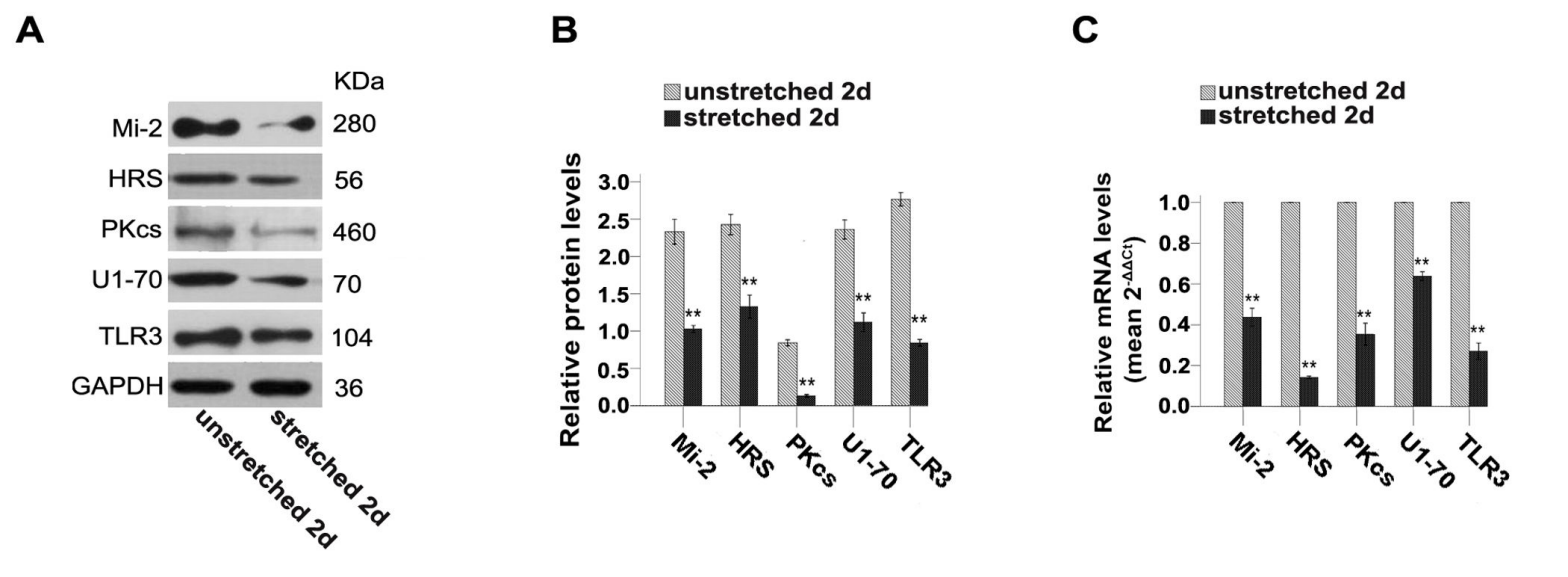
Mechanical-stretch inhibits the levels of autoantigens and of TLR3 in C2C12 cells. (**A**) Western blots analysis showing the immune detection of proteins that can serve as autoantigens (Mi-2, HRS, DNA-PKcs, U1-70) and of TLR3. (**B**) The relative band intensities from western blots experiments were normalized to the level of GAPDH and analyzed with Quantity One software. (**C**) mRNA levels corresponding to proteins that can serve as autoantigens and TLR3 were evaluated in 2d stretched and unstretched cells by qRT-PCR. One-way ANOVA was used for multiple comparisons. All data are presented as mean ± SD (n=3). ( **p<0.001).

Cultured human primary myoblasts express high levels of autoantigens, which are strikingly down regulated as cells differentiate into myotubes *in vitro* [15]. This would imply that resistance training, by inducing muscle regeneration cycles, could also be associated with their transient up-regulations. Therefore, we thought to evaluate here in our *in vitro* model how potential autoantigens are regulated in stretched C2C12 myoblasts. For that, cycles of mechanical strain were applied during 2 days and the expression levels of classical muscle autoantigens (i.e., Mi-2, HRS, DNA-PKcs and U1-70) and of the proinflammatory TLR3 and TLR7 were determined by real time qRT-PCR and by immunoblotting. Results showed that mRNA and protein levels of Mi-2, HRS, DNA-PKcs and U1-70 were unexpectedly decreased in stretched cells as compared to unstretched controls (Figure 3). 

Because TLR3 and TLR7 are involved in chronic muscle inflammation and are expressed mainly by immature regenerating myofibres and in less differentiated myoblasts *in vitro* [16], we sought also to examine the effects of mechanical strain on TLR3 and TLR7 expression in C2C12 myoblasts. Remarkably, we found that TLR3 expression, akin to autoantigens expression, was also inhibited following 2 days of mechanical-stretch (Figure 3). We could not study how TLR7 expression is regulated by strain, as we did not detect corresponding mRNA or protein in C2C12 myoblasts (data not shown).

### Agonist or antagonist of the key molecules involved in the response to mechanical-stretch interfere with the expression of autoantigens and TLR3 in C2C12 myoblasts

To further investigate the mechanisms involved in the down regulation of autoantigens and of TLR3 in stretched C2C12 myoblasts, we next evaluated the effects of competitive inhibitors, or conversely of agonists, targeting the key molecules involved in the response to mechanical-stretch (i.e., calmodulin, MMP-2, HGF, c-Met and nNOS). For that, C2C12 cells were first cultured for 24h before adding selected inhibitors or agonists during 4h in the absence of any mechanical-stretch. Expression of autoantigens and TLR3 were then evaluated, as in previous experiments, by qRT-PCR and by Western Blotting.

**Figure 4:**
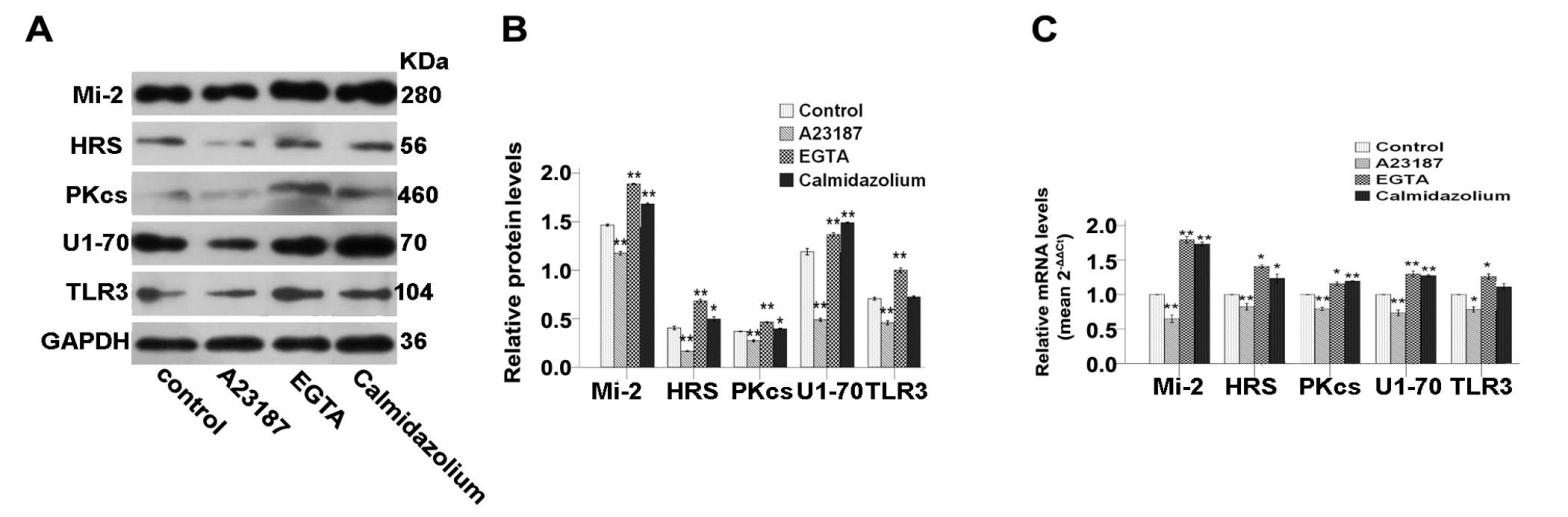
Agonist and antagonist of the calcium/calmodulin pathway interfere with the expression of autoantigens and of TLR3 in C2C12 cells. Unstretched C2C12 cells were cultured for 24h and then treated with 3μM A23187, 1.8mM EGTA, or 0.3μM calmidazolium chloride during 4h. (**A**) Western blots analysis showing the immune detection of proteins that can serve as autoantigens and of TLR3 in treated and untreated cells. (**B**) The relative band intensities from western blots experiments were normalized to the level of GAPDH and analyzed with Quantity One software. (**C**) mRNA levels corresponding to that can serve as autoantigens and of TLR3 were quantified by qRT-PCR analysis in treated or untreated cells. One-way ANOVA was used for multiple comparisons. All data are presented as mean ± SD (n=3). (**p<0.001; *p<0.05).

We first evaluated the involvement of the calcium-binding calmodulin protein in the modulation of autoantigens and TLR3 expression. For that, C2C12 myoblasts were treated with molecules that could either stimulate its activity (i.e., the calcium ionophore A23187) or, conversely, molecules that could inhibit its activity (i.e., the calcium chelator EGTA or the more specific calmodulin inhibitor calmidazolium). Results showed that the A23187 calcium ionophore indeed mimicked the effect of mechanical-stretch and inhibited autoantigens and TLR3 expression (Figure 4). Inversely, chelation of calcium by EGTA or treatment with the calmidazolium increased levels of most proteins at study (Figure 4).

**Figure 5:**
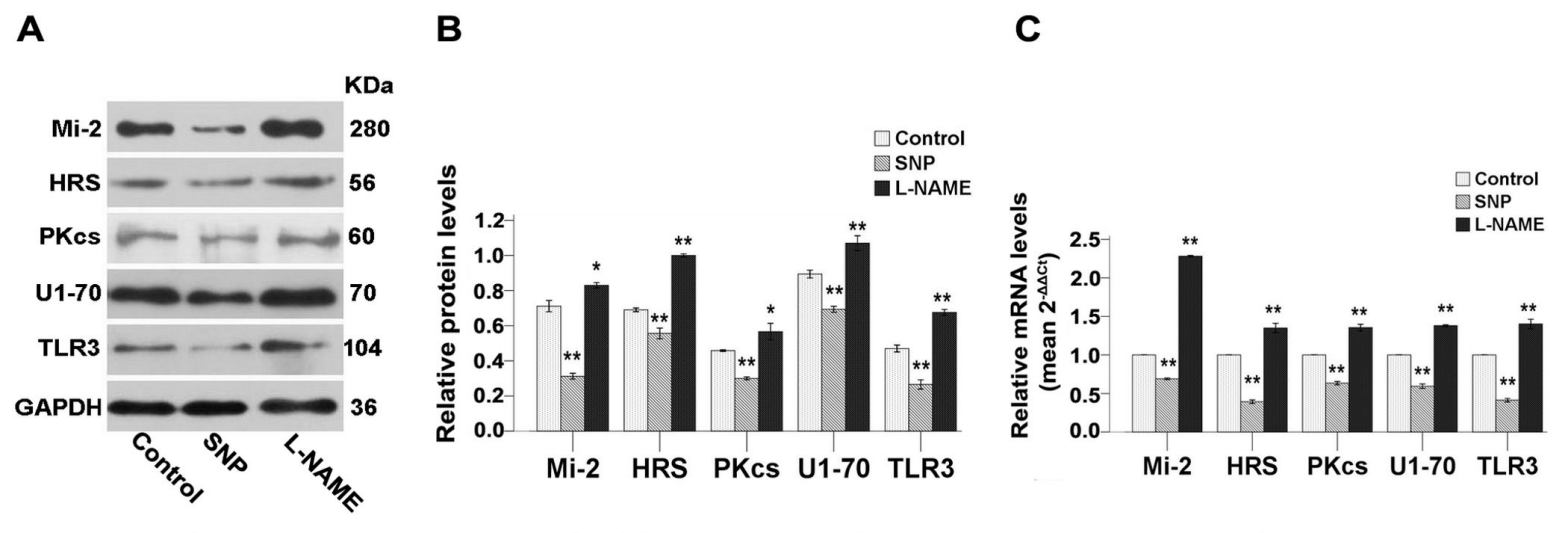
NO donor and antagonist of NOS activity interfere with the expression of autoantigens and of TLR3 in C2C12 cells. Unstretched C2C12 cells were cultured for 24h and then treated with 30μM SNP (NO donor) or 10μM L-NAME (inhibitor of NOS activity) during 4h. (**A**) Western blots analysis showing the immune detection of proteins that can serve as autoantigens and of TLR3 in treated and untreated cells. (**B**) The relative band intensities from western blots experiments were normalized to the level of GAPDH and analyzed with Quantity One software. (**C**) mRNA levels corresponding to proteins that can serve as autoantigens and of TLR3 were quantified by qRT-PCR analysis in treated or untreated cells. One-way ANOVA was used for multiple comparisons. All data are presented as mean ± SD (n=3). (**p<0.001; *p<0.05).

Next, we similarly explored the involvement of NOS and of its enzymatic product NO by using SNP (a donor of NO), or L-NAME (an inhibitor of nitric oxide synthesis). Results showed that the expression of autoantigens and TLR3 were down regulated by the NO donor SNP and, conversely, upregulated by inhibition of the production of NO using L-NAME (Figure 5). 

**Figure 6:**
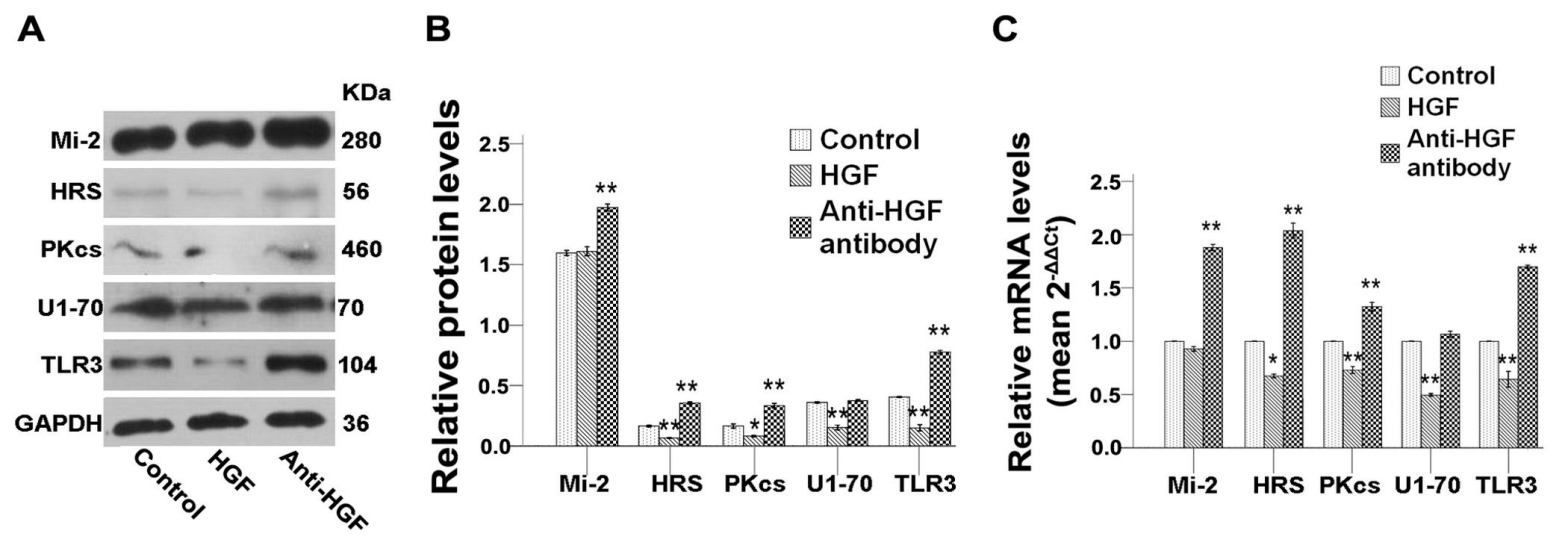
HGF and anti-HGF interfere with the expression of autoantigens and of TLR3 in C2C12 cells. Unstretched C2C12 cells were cultured for 24h and then treated with 20ng/ml recombinant murine HGF or 2μg/ml anti-HGF antibody. (**A**) Western blots analysis showing the immune detection of proteins that can serve as autoantigens and of TLR3 in treated and untreated cells. (**B**) The relative band intensities from western blots experiments were normalized to the level of GAPDH and analyzed with Quantity One software. (**C**) mRNA levels corresponding to proteins that can serve as autoantigens and of TLR3 were quantified by qRT-PCR analysis in treated or untreated cells. One-way ANOVA was used for multiple comparisons. All data are presented as mean ± SD (n=3). (**p<0.001; *p<0.05).

**Figure 7:**
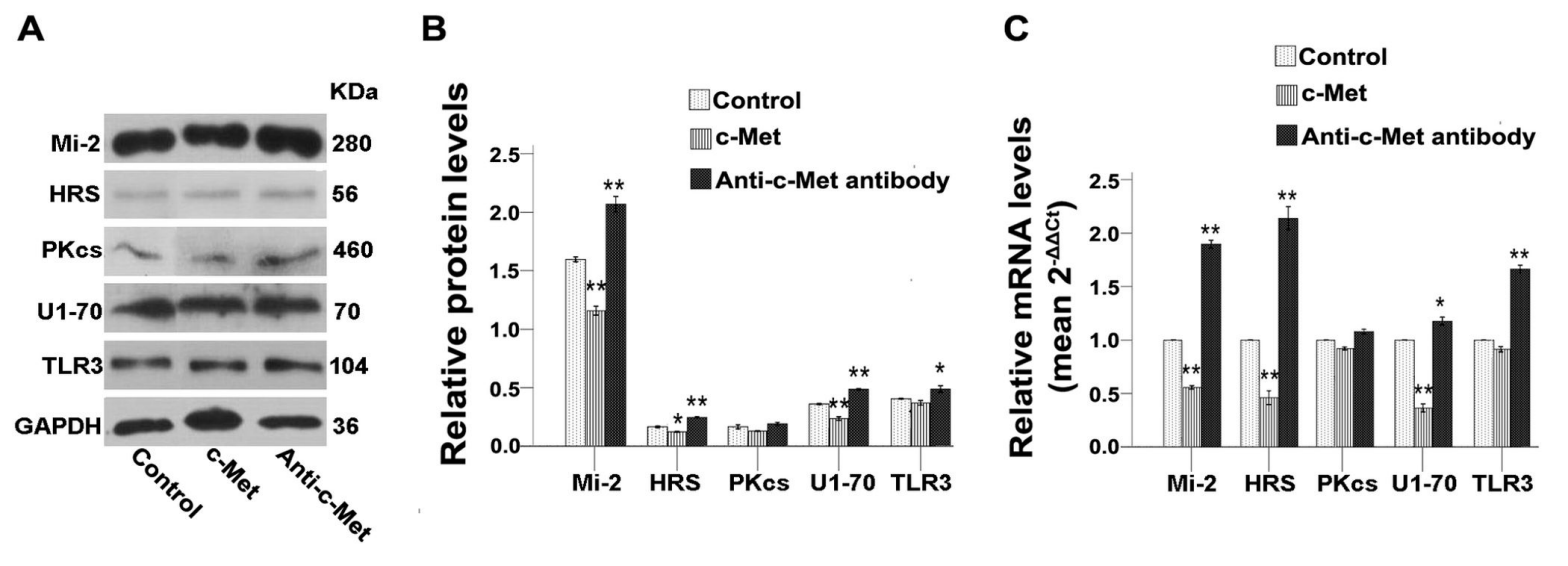
c-Met and anti-c-Met antibody interfere with the expression of autoantigens and of TLR3 in C2C12 cells. Unstretched C2C12 cells were cultured for 24h and then treated with 1μg/ml recombinant mouse c-Met or 2μg/ml anti-c-Met antibody. (**A**) Western blots analysis showing the immune detection of proteins that can serve as autoantigens and of TLR3 in treated and untreated cells. (**B**) The relative band intensities from western blots experiments were normalized to the level of GAPDH and analyzed with Quantity One software. (**C**) mRNA levels corresponding to proteins that can serve as autoantigens and of TLR3 were quantified by qRT-PCR analysis in treated or untreated cells. One-way ANOVA was used for multiple comparisons. All data are presented as mean ± SD (n=3). (**p<0.001; *p<0.05).

In following experiments, the roles of HGF and c-Met were explored by adding directly these proteins in culture, or by adding instead anti-HGF or anti-c-Met blocking antibodies. Results showed a significant inhibition of the expression of almost all autoantigens studied here and of TLR3 when C2C12 cells were treated with recombinant HGF and c-Met proteins (Figure 6 and Figure 7). Conversely, treatments with anti-HGF or anti-c-Met antibodies resulted in the upregulation of most of these proteins (Figure 6 and Figure 7). Of notes, adding MMP-2 recombinant protein *in vitro* did not support a direct role of MMP-2 in the regulation of autoantigens and TLR3 expression (data not shown). Collectively our results showed that stimulation or inhibition of calmodulin, NOS, HGF or c-Met molecules *in vitro* affected the expression of autoantigen and TLR3 proteins supporting the involvement of this molecular pathway in the inhibition of autoantigens and TLR3 observed upon application of mechanical-stretch. 

## Discussion

Expression of TLR3 and of proteins that could serve as autoantigens is readily detected in biopsies from patients suffering from inflammatory myopathies and may contribute to the pathophysiology of autoimmune myositis [15-17]. In such patients, resistance training was not recommended as it could supposedly aggravate the symptoms by inducing muscle regeneration and consequently the upregulation of these proteins expressed at higher levels in regenerating fibers and in myoblasts. However, recent data have suggested instead that physical activity could be beneficial in patients with myositis by reducing systemic inflammation and fibrosis [11,12]. Conceivably, moderate exercise may exert its newly recognized beneficial role partly through the induction of key mechanical-strain responsive molecules culminating in the activation of satellite cells and in muscle repair. However, how such a mechanism would influence the expression of potential autoantigens and of TLRs has not yet been explored. Therefore, the aim of our study here was to evaluate the consequences of mechanical-stretch on, i) the stimulation of proliferation and cell cycling, ii) the expression of genes related to the mechanical-stretch pathway (e.g., genes coding for calmodulin, nNOS, MMP-2, HGF and c-Met) and, iii) the expression of TLRs and of proteins that could serve as autoantigens. We showed here that cyclic mechanical-stretch stimulated C2C12 cell cycling but also the early up-regulation of the molecules related to the mechanical-stretch pathway in muscle (calmodulin, nNOS, MMP-2, HGF and c-Met). Unexpectedly, mechanical stretch also reduced the expression of TLR3 and of proteins known to represent autoantigens in inflammatory autoimmune myopathies (Mi-2, HRS, PKcs, U1-70).

Specific autoantigens targeted by the immune system in autoimmune myositis patients have been well characterized. They include Mi-2, histidyl tRNA synthetase (HRS/Jo-1), U1-70kD, or the catalytic subunit of DNA-dependent protein kinase (DNA-PKcs) [17,22]. Interestingly, in biopsies obtained from patients suffering from myositis, the proteins corresponding to these autoantigens as well as the potentially proinflammatory TLR3 and TLR7 receptors were found to be up-regulated in regenerating myoblasts [15,16,22]. Higher expression of these proteins could also be demonstrated in primary satellite cells cultured *in vitro* as compared to differentiated myotubes [13,15,16]. We show here that these proteins are also expressed, although at different levels, in cultured C2C12 cells. In our assays, we seeded C2C12 cells at a low cellular density and cultivated them for less than 4 days to avoid confounding effects of cell fusion and cell differentiation. Among proteins that could serve as autoantigens, we found that DNA-PKcs level was expressed at the lowest levels as compared to Mi-2, HRS or U1-70 (Figure 3). Whatever their basal level in control cells, we conspicuously observed a significant down-regulation of the expression of Mi-2, HRS, U1-70, DNA-PKcs and TLR3 in C2C12 cells stretched during 2d (Figure 3). This suggests that myoblasts are directly sensitive to mechanical-stretch which rapidly stimulates their entry into cell cycle and concomitantly inhibits the expression of potential autoantigens and of TLR3 (Figures 1 and 3).

How does mechanical strain applied *in vitro* influence C2C12 cells and could our model mimic moderate exercise? While setting up the model we found that cells submitted to a 15%, 1Hz, 1h/d stretching protocol detached readily and showed signs of cell death. This is consistent with previous report showing that cell lengthening set to levels≧15% causes significant cell death [20,21]. Instead, mechanical-stretch of 10% was suggested to more closely mimic the mechanical strain that could be induced by moderate exercise without provoking cell injury [20,21]. Our data are compatible with this notion as a 10%, 0.25Hz, 2h/d stretching protocol did not cause detachment of myoblast and stimulated instead their entry into cell cycle (Figure 1). The molecular cascade involved in the response of satellite cells to mechanical strain has been recently described and involved calcium signaling, calmodulin activation, production of NO, activation of MMP-2, liberation of HGF and stimulation of c-Met receptor [10]. We could demonstrate here that *in vitro* stretched C2C12 cells also responded to mechanical-stretch in a similar fashion by up-regulating calmodulin, nNOS, MMP-2, HGF and c-Met (Figure 2). Furthermore, we showed here that manipulating this pathway using agonists/antagonists could also regulate autoantigens and TLR3 levels (Figures 4-7). This further confirmed the role of key molecules involved in the response to mechanical-stretch and suggested their direct role in this phenomenon. 

The precise molecular mechanism by which mechanical strain regulates autoantigens and TLR3 levels is not clear. It is tempting to speculate that modulation of gene expression of autoantigens and TLR3 is directly controlled by the molecules upregulated by mechanical-stretch. Notably, calcium influx and activation of calmodulin, production of NO, and stimulation of HGF/c-Met pathway were capable here to directly modulate the expression of these genes when manipulated *in vitro*. Interestingly, calcium/calmodulin-dependent pathway has been implicated in regulating skeletal muscle gene expression [23,24]. Also, HGF represents a multi-functional cytokine that stimulates mitogenesis, cell motility, matrix invasion, tissue regeneration, and is known also to reduce chemokine genes expression [25].

Apart from stimulation of myoblast proliferation and from down regulation of autoantigens and TLR3 levels, mechanical strain may conceivably induce different mechanisms that may collectively explain its beneficial role. For instance, a transgenic mouse model over-expressing muscle-specific nNOS suggested its anti-inflammatory role *in vivo* by preventing neutrophil-mediated muscle injury [26]. Also, HGF release stimulates mitogenesis, prevents fibrosis and also suppresses antigen-specific immune responses in part by suppressing dendritic cell function and chemokine expression [25,27-29]. Thus, up regulation of HGF upon injury, may both stimulate muscle regeneration as well as locally suppress immune reactions. 

Collectively the data presented here show that mechanical strain stimulates the proliferation of myoblasts cultured *in vitro* and the production of proteins involved in the mechanical-stretch pathway (i.e., calmodulin, nNOS, MMP-2, HGF and c-Met). Moreover, mechanical strain also down regulated expression of proteins that can serve as autoantigens and, additionally, of the proinflammatory TLR3 receptor. Even if our *in vitro* data will still need to be confirmed in the pathophysiological context, our results so far suggest that moderate exercise may be beneficial in patients suffering from myopathies by stimulating muscle regeneration and, possibly also, by limiting the availability of immune stimulating molecules. Our data also suggest that pharmacological manipulation of the key molecules involved in the response to mechanical-stretch may partly mimic the beneficial effect of exercise training.
